# EMG-Informed Neuromusculoskeletal Simulations Increase the Accuracy of the Estimation of Knee Joint Contact Forces During Sub-optimal Level Walking

**DOI:** 10.1007/s10439-025-03713-2

**Published:** 2025-03-24

**Authors:** Domitille Princelle, Marco Viceconti, Giorgio Davico

**Affiliations:** 1 Department of Industrial Engineering, Alma Mater Studiorum - University of Bologna, Bologna, Italy; 2 Medical Technology Lab, IRCCS Istituto Ortopedico Rizzoli, Bologna, Italy

**Keywords:** Subject-specific models, Neuromusculoskeletal models, Joint contact forces, EMG-informed simulation, Predictive accuracy

## Abstract

**Purpose:**

Personalized musculoskeletal models are crucial to get insights into the mechanisms underpinning neuromusculoskeletal disorders and have the potential to support clinicians in the daily management and evaluation of patients. However, their use is still limited due to the lack of validation studies, which hinders people’s trust in these technologies. The current study aims to assess the predictive accuracy of two common approaches to estimate knee joint contact forces, when employing musculoskeletal models.

**Methods:**

Subject-specific musculoskeletal models were developed for four elderly subjects, exploiting the freely accessible Knee Grand Challenge datasets, and used to perform biomechanical simulations of level walking to estimate knee joint contact forces. The classical static optimization and EMG-assisted approaches were implemented to resolve the muscle redundancy problem. Their estimates were compared, in terms of predictive accuracy, against the experimental recordings from an instrumented knee implant and against one another. Spatiotemporal differences were identified through Statistical Parametrical Mapping, to complement traditional similarity metrics (*R*^2^, RMSE, 95th percentile, and the maximal error).

**Results:**

Both methods allowed to estimate the experimental knee joint contact forces experienced during walking with a high level of accuracy (*R*^2^ > 0.82, RMSE < 0.56 BW). The EMG-assisted approach further enabled to highlight subject-specific features that were not captured otherwise, such as a prolonged or anticipated muscle-co-contraction.

**Conclusion:**

While the static optimization approach provides reasonable estimates for subjects exhibiting typical gait, the EMG-assisted approach should be preferred and employed when studying clinical populations or patients exhibiting abnormal walking patterns.

**Supplementary Information:**

The online version contains supplementary material available at 10.1007/s10439-025-03713-2.

## Introduction

Neuromusculoskeletal (MSK) conditions (e.g., osteoarthritis, stroke, cerebral palsy) contributed—in 2019—to nearly 65% of the total burden of diseases, expressed in terms of years lived with disability [1]. According to the 2019 Global Burden of Diseases, Injury, and Risk Factors Study, this figure has not significantly changed since 1999 [1]. The reason can be found in the standardization of most rehabilitation programs, treatment designs, and medical interventions that tend to disregard individual physiological differences. These may not be easily captured by traditional clinical tests but may become apparent when observing internal biomechanical quantities, such as muscle and intersegmental forces. The latter cannot be readily measured in vivo but can be predicted, with MSK models, and may provide insights into the motor control strategies selected by an individual while performing various activities of daily living, thus highlighting any ongoing compensatory mechanisms or abnormal muscle activation patterns (suboptimal muscle control) [2]. In addition, the investigation of articular joint loads may enable the identification of the onset and progression of joint degenerative diseases, such as osteoarthritis [3]. Clinicians and bioengineers should work more closely together to tailor the clinical management (from diagnosis to treatment and rehabilitation) to the patient’s needs. To this end, the development and use of patient-specific MSK models are instrumental [4, 5].

Patient-specific MSK models are being employed to investigate gait kinematics, kinetics, and intersegmental forces during various locomotor tasks [6–9]. However, due to the length and complexity of the pipelines and optimization approaches required to develop these models [4, 10, 11], their use in clinical practice is still limited. This problem was partly addressed by the introduction of specialized software tools, such as the freeware nmsBuilder [12] and the STAPLE toolbox [13], and by the definition of guidelines and pipelines (e.g., the INSIGNEO pipeline [10]) to standardize the workflow. In a recent study, we proposed and validated a modeling pipeline that reduced the average time to generate one of such models from days to hours [14]. What remains to be thoroughly assessed (on multiple subjects) is the predictive accuracy of patient-specific MSK models, especially considering the numerous approaches to model neuromuscular control [15]. Previous validation works were limited in the number of subjects and trial studied (typically one or two [16]).

There is evidence in the literature showing that healthy (adult) individuals tend to select a neuromuscular activation strategy that minimizes the metabolic expenditure when performing sub-maximal stereotypical tasks, such as level walking [17, 18]. This strategy, sometimes referred to as *optimal control* [19–21], is commonly predicted using a static optimization (SO) algorithm [22] that minimizes the sum of squared muscle activations [23]. When the neuromuscular control is mildly suboptimal, one can assume that the deviation from the optimal control is due to the so-called *uncontrolled manifold* theory [24, 25]; this makes it possible to still describe the control in terms of optimal control but in a stochastic sense. On the other hand, when the subject control is severely sub-optimal, the only viable solution so far is to resort to surface electromyography (EMG) data—if available—to inform the simulations. EMG-informed models select the neuromuscular control strategy leveraging on information from the surface potentials evoked by a few relevant muscles, as measured by surface EMG sensors [7, 26–32].

This study aims to assess the predictive accuracy of patient-specific models generated with our pipeline [14] by comparing the total knee joint contact force as predicted by the model to the value measured by an instrumented prosthesis acquired in four editions of the Knee Grand Challenge competition [33]. In addition, this study aims to investigate how the predictive accuracy would change when the EMG-informed approach replaces the conventional SO approach.

## Materials and Methods

### Experimental Data

The experimental data employed in this study belong to the last four editions of the Grand Challenge competition [33]. Two other datasets exist and correspond to the first and second Challenges but were not used as subsequent (more recent) editions collected data on the same participants. All participants wore an instrumented knee implant that enabled the *in vivo* recording of knee loads. In addition, motion capture, ground reaction force, and EMG data from the lower limb muscles were simultaneously collected (Table 1). Furthermore, pre- and post-operative medical imaging data of the lower limb of interest, including the 3D reconstructions of the prosthetic components and some bony geometries, were provided. Missing geometries, such as foot and calcaneus geometry, were extracted from a generic scaled full-body model [34], and incomplete pelvic geometries (e.g., hemipelvis) were reconstructed in the MAP client employing statistical shape modeling techniques [35, 36].

**Table 1:** Patients’ demographics and experimental data used, including EMG quality check.

Grand challenge edition	3rd	4th	5th	6th
Gender	Female	Male	Male	Male
Mass (kg)	78.4	66.7	75	70
Gait trials (#)	5-6-7-8	2-3-4-5-7	1-8-9-11	3-4-5-6-7-9
Muscles EMG				
Vastus lateralis	V	V	V	V
Vastus medialis	X	Noise	V	Noise
Tibialis anterior	X	V	V	V
Biceps femoris long head	V	V	V	Noise
Semimembranosus	V	V	V	V
Lateral gastrocnemius	V	V	V	V
Medial gastrocnemius	V	V	V	V
Gluteus maximus	V	V	Noise	V
Gluteus medius	X	Noise	Noise	Noise
Adductor magnus	V	V	V	V
Rectus femoris	V	V	V	V
Peroneus longus	X	Noise	V	V
Soleus	X	X	V	V
Sartorius	X	X	V	X

### Data Processing

For each participant, motion capture data were processed in MATLAB® (R2021b, The MathWorks Inc., Massachusetts) using MOtoNMS [37]. Specifically, marker trajectories and ground reaction forces were low pass filtered with a zero-lag 4th-order Butterworth filter (*f*_LP_ = 8 Hz). Then, the EMG signals were band-pass filtered between 30 and 300 Hz [38], full wave rectified, and low pass filtered at 8 Hz using a 4th-order zero-lag Butterworth filter. The filtered EMG data were subsequently amplitude normalized to the maximum value observed in the available overground walking trials [37, 38]. Experimental EMG data were discarded if the signals appeared noisy or faulty, in line with previous work [39] (Table 1). The number of useable trials varied between datasets: four trials for the 3rd and 5th editions of the challenge and five for the 4th and six for the 6th challenges (Table 1).

### Musculoskeletal Models

**Fig. 1:**
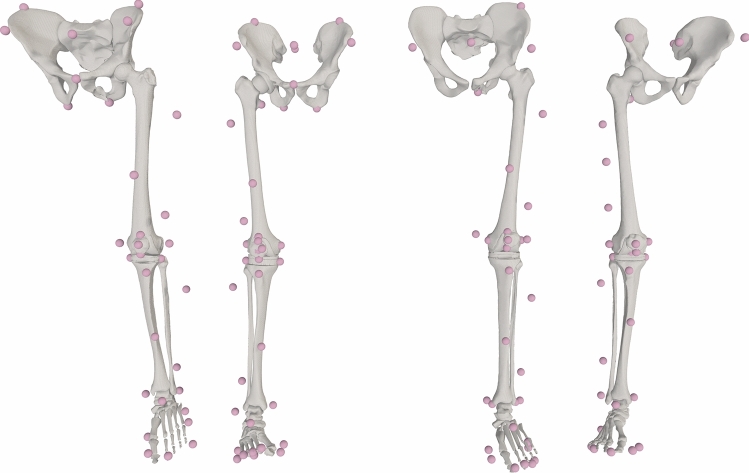
Image-based musculoskeletal models employed in the study. From left to right, the models for KGC3, KGC4, KGC5, and KGC6. All muscle pathways have been hidden for clarity.

For all patients, a single-leg patient-specific MSK models was generated following our pipeline [14] that relies on the STAPLE toolbox [13] and the nmsBuilder freeware [12]. Each model included 7 segments, 13 degrees of freedom, and 40 muscle actuators and featured a patellofemoral joint that was coupled to the knee joint [34] (Fig. 1).

The tendon slack and optimal fiber lengths, derived from the generic full-body model [34], were morphometrically scaled [40]. The maximal isometric force (MIF) values were personalized exploiting the available CT data. Specifically, for each muscle, the MIF was scaled by the ratio between the total lower limb muscle volume extracted from the CT scans and the corresponding volume from the generic whole-body model [34], which was back calculated from the generic muscle parameters. When the accuracy of the CT scan permitted, the muscles were individually segmented and personalized using the PCSAs. Of note, to personalize the model of the subject enrolled in the 3rd edition of the challenge, who was a 68-year-old woman, data from an open access database on postmenopausal women were exploited [41].

### Biomechanical Simulation

Within OpenSim 4.1 [42, 43], the four patient-specific MSK models were employed to run biomechanical simulations of gait to predict the tibiofemoral contact forces. The Inverse Kinematics tool, which reconstructs model motion from marker data, was used to compute joint coordinates. Following, the external joint torques were predicted using the recursive Newton–Euler method within the inverse dynamics tool, and the muscle moment arms, muscle–tendon lengths, and velocities were computed through a Muscle Analysis. From there, two approaches were used to solve the muscle redundancy problem and estimate muscle forces and activations: static optimization (SO) and EMG-assisted approaches.

The static optimization approach enabled to identify the solution corresponding to the minimal energy expenditure (minimized metabolic cost). At each frame, the solver identified the solution that minimized the overall sum of squared muscle activations [22, 23], while satisfying the dynamic equilibrium and respecting the physiological bounds (i.e., tetanic muscle force defined by the maximal isometric force values) as per equation below:

$$F_{{{\text{obj}}}} = \mathop \sum \limits_{{j \in {\text{MTUs}}}} \left({a}_{j} \right)^{2},$$

where *a*_j_ is the j-th muscle activation.

The force–length–velocity relationship was enforced, and, to resolve residual dynamic inconsistencies (between the multi-body dynamics and the experimental data) and numerical errors, ideal torque actuators (residual and reserve actuators) were added to the model to assist muscles when the latter (alone) could not balance the external torques. However, to discourage the use of the reserve actuators, thus minimizing their contribution to the motion, their optimal force was set to 1 Nm.

Following, EMG-assisted simulations were performed, exploiting the Calibrated EMG-Informed Neuromusculoskeletal Modeling Toolbox (CEINMS) [44]. The models were first calibrated, i.e., the muscle parameters were tuned leveraging on the available experimental EMG data to minimize both joint torques and EMG tracking errors [7, 32]. During calibration, the tendon slack and optimal fiber lengths were allowed to vary by 10% from their original values, while the strength coefficient (which acts as force multiplier) was allowed to vary between 0.8 and 1.3. Model calibration was conducted using one walking trial per participant, while the remaining gait trials were left for the analyses. Of note, within CEINMS, the model parameters defined the conversion of basic EMG into muscle excitation and in turn into muscle activation via activation dynamics, and muscle force via the Hille-type muscle–tendon model.

EMG-assisted simulations were then performed on the calibrated models, which all featured 5 DOFs (hip flexion, adduction and rotation, knee flexion, and ankle plantar/dorsiflexion). The following cost function was solved at each time frame to solve the muscle redundancy problem:

$$F_{{{\text{obj}}}} = \alpha *\mathop \sum \limits_{{k \in {\text{DOFs}}}} \left( {\tau_{k} - \tilde{\tau }_{k} } \right)^{2} + \beta *\mathop \sum \limits_{{j \in {\text{MTUs}}}} \left( {\tilde{e}_{{\text{j}}} } \right)^{2} + \gamma *\mathop \sum \limits_{{j \in {\text{MTUs}}}} \left( {e_{{\text{j}}} - \tilde{e}_{{\text{j}}} } \right)^{2},$$

where the first and last terms refer—respectively—to the joint torques and EMG tracking errors between predicted and experimental values, while the second term is to minimize the overall sum of squared excitations (similarly to the static optimization approach). The three coefficients (α, β and γ), weighting each term, were set to 1, 10, and 20.

Once muscle forces were estimated, with both approaches, the joint contact forces were estimated using the Joint Reaction Analysis tool, exploiting the OpenSim API for MATLAB (v2021b).

### Data Analysis

All data (i.e., both experimental and predicted) were interpolated to 101-point vectors to be expressed as a percentage of the gait cycle (0–100%), to facilitate the analyses and to enable the separation between stance and swing phase. In addition, to allow for comparisons between subjects, the joint torques data were normalized to each participant’s mass, while the predicted joint contact forces were normalized by the body weight (BW). Similarly, the recordings of the instrumented implants were converted into a total contact force expressed in the tibial reference system and amplitude normalized to BW.

For the EMG-assisted approach, the joint torques and EMG tracking errors (between corresponding experimental and simulated profiles) were computed by calculating the coefficient of determination (*R*^2^) and the root mean-squared error (RMSE), as measures of similarity between curves. Of note, the trials employed in the calibration process (one per subject) were excluded from this analysis.

For each subject and trial by trial, the normalized knee joint contact forces predicted by the models employing the two tested approaches (SO and EMG-assisted) were compared to corresponding experimentally recorded data (*implant*) using four different metrics, i.e., R^2^, RMSE, 95th percentile, and maximal error (Error_Max_). This analysis was repeated twice: once considering the entire trial and once on the stance phase only.

In addition, using a non-parametric two-tailed *t* test [SnPM{*t*} with *α* = 0.05, [45]], a temporal statistical analysis was conducted to highlight when in the gait cycle, for each participant, the estimated total knee contact forces and the measured values were significantly different from one another (*p* < 0.05).

## Results

**Fig. 2:**
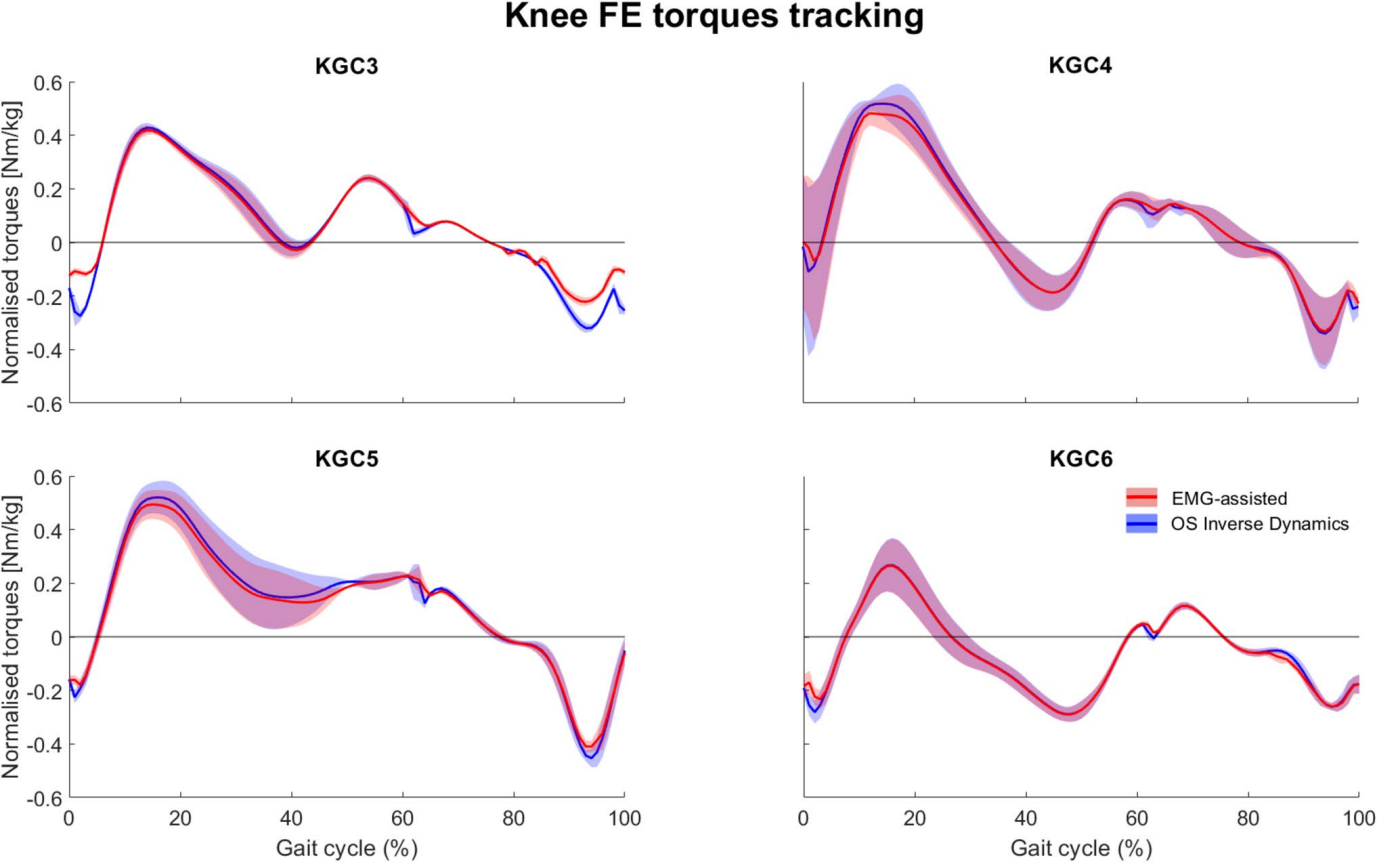
Knee joint moments tracking error. Knee flexion/extension moments predicted by the models within OpenSim (inverse dynamics, red line) and CEINMS (blue line), for all four KGC competitions. Data are reported as mean and standard deviation across trials.

For all participants, the models proved able to accurately track the experimental motion capture data, as demonstrated by the low RMS and maximal errors, well within the OKAY thresholds set by the OpenSim best practices (Table S1, Supplementary Information). Similarly, the CEINMS-calibrated models properly tracked the experimental joint torques predicted by the OpenSim’s inverse dynamics tool, with RMSE < 0.25 Nm/kg and *R*^2^ > 0.95 for all cases and degrees of freedom, but the hip rotation for KGC4 (Fig. 2 and Table S2, Supplementary Information).

Overall, both the static optimization and EMG-assisted approaches enabled to predict total knee joint contact forces in line with those recorded by the instrumented implant (*R*^2^ > 0.82, RMSE < 0.56 BW), with some distinctions. Independently on the selected approach, the predictive accuracy of the models was higher for KGC3 and KGC5, than for KGC4 and KGC6 participants (static optimization: *R*^2^ = 0.92 ± 0.02 for KGC3, 0.83 ± 0.03 for KGC4, 0.95 ± 0.01 for KGC5 and, 0.89 ± 0.04 for KGC6; EMG-assisted approach: *R*^2^ = 0.93 ± 0.03 for KGC3, 0.91 ± 0.02 for KGC4, 0.96 ± 0.00 for KGC5 and, 0.92 ± 0.02 for KGC6) (Table 2).

**Table 2:** Predictive accuracy across the whole gait cycle

Participant	Trials	*R*^2^		RMSE (BW)		95th percentile (BW)		Error_Max_ (BW)	
		SO	EMGa	SO	EMGa	SO	EMGa	SO	EMGa
KGC3	ngait_og5	0.91	–	0.32	–	0.60	–	0.64	–
	ngait_og6	0.94	**0.97**	0.28	**0.23**	0.57	0.54	0.63	0.63
	ngait_og7	0.91	**0.92**	**0.34**	0.35	0.57	0.67	**0.64**	0.87
	ngait_og8	**0.94**	0.90	**0.29**	0.38	0.65	0.88	**0.75**	1.02
KGC4	ngait_og2	0.86	**0.91**	0.41	**0.38**	0.81	0.64	0.95	**0.91**
	ngait_og3	0.85	**0.88**	**0.41**	0.42	0.81	0.86	0.91	0.91
	ngait_og4	0.78	**0.91**	0.49	**0.32**	0.93	0.58	1.15	**0.65**
	ngait_og5	0.82	–	0.41	–	0.77	–	0.96	–
	ngait_og7	0.85	**0.93**	0.47	**0.34**	0.89	0.62	1.19	**0.71**
KGC5	ngait_og1	0.95	–	0.29	–	0.56	–	0.67	–
	ngait_og8	0.96	0.96	0.29	**0.27**	0.58	0.56	0.89	**0.70**
	ngait_og9	0.95	**0.96**	**0.31**	0.33	0.49	0.69	**0.52**	0.80
	ngait_og11	0.96	0.96	**0.24**	0.32	0.50	0.69	**0.52**	0.80
KGC6	ngait_og3	0.88	–	0.48	–	0.95	–	1.07	–
	ngait_og4	**0.95**	0.93	**0.26**	0.42	0.53	0.87	**0.67**	1.00
	ngait_og5	0.93	0.93	0.36	**0.28**	0.68	0.52	0.86	**0.72**
	ngait_og6	0.84	**0.91**	0.56	**0.39**	1.25	0.75	1.58	**0.85**
	ngait_og7	0.85	**0.92**	0.48	**0.36**	1.12	0.90	**1.20**	1.33
	ngait_og9	0.90	0.90	**0.40**	0.42	0.83	0.82	**0.97**	1.09

Comparisons between experimental data and corresponding knee joint contact force predicted using static optimization and EMG-assisted approaches, across the entire gait cycle, in terms of *R*^2^, RMSE, 95th percentile, and maximal error (Error_Max_).

When considering the whole gait cycle, in general (13 out of 15 trials), the EMG-assisted approach allowed to equally or better approximate the experimental total knee joint contact forces compared to static optimization (bold *R*^2^ values, Table 2). On the other hand, neither approach emerged as clear best in terms of RMSEs (Table 2). However, when limiting the analyses to the stance phase only, the EMG-assisted approach showed to yield more accurate predictions than static optimization, both in terms of *R*^2^ and RMSEs (Table 3).

**Table 3:** Predictive accuracy across the stance phase of the gait cycle

Participant	Trials	*R*^2^		RMSE (BW)		95th percentile (BW)		Error_Max_ (BW)	
		SO	EMGa	SO	EMGa	SO	EMGa	SO	EMGa
KGC3	ngait_og5	0.78	–	0.34	–	0.60	–	0.64	–
	ngait_og6	0.87	**0.94**	0.32	**0.26**	0.59	0.59	0.63	0.63
	ngait_og7	0.83	**0.85**	0.34	**0.32**	0.57	0.71	**0.64**	0.87
	ngait_og8	**0.84**	0.75	**0.34**	0.46	0.67	0.96	**0.75**	1.02
KGC4	ngait_og2	0.70	**0.93**	**0.41**	0.43	0.74	0.78	**0.83**	0.91
	ngait_og3	0.71	**0.74**	**0.46**	0.50	0.81	0.89	**0.83**	0.91
	ngait_og4	0.47	**0.79**	0.51	**0.35**	0.92	0.62	0.94	**0.65**
	ngait_og5	0.58	–	0.45	–	0.81	–	0.96	–
	ngait_og7	0.67	**0.83**	0.52	**0.37**	0.87	0.67	0.91	**0.71**
KGC5	ngait_og1	0.89	–	0.27	–	0.55	–	0.62	–
	ngait_og8	0.88	**0.93**	0.34	**0.21**	0.74	0.46	0.89	**0.67**
	ngait_og9	0.88	**0.94**	0.33	**0.25**	0.46	0.50	**0.49**	0.57
	ngait_og11	0.88	**0.90**	0.23	**0.21**	0.44	0.44	**0.50**	0.52
KGC6	ngait_og3	0.65	–	0.53	–	1.00	–	1.07	–
	ngait_og4	**0.88**	0.78	**0.28**	0.46	0.53	0.89	**0.67**	0.99
	ngait_og5	**0.81**	0.76	0.40	0.40	0.70	0.82	0.86	**0.84**
	ngait_og6	0.50	**0.67**	0.67	**0.53**	1.41	0.97	1.58	**0.98**
	ngait_og7	0.66	**0.73**	0.56	**0.51**	1.14	1.25	**1.20**	1.39
	ngait_og9	**0.80**	0.60	**0.47**	0.62	0.87	1.30	**0.97**	1.53

Comparisons between experimental data and corresponding knee joint contact force predicted using static optimization and EMG-assisted approaches, across the stance phase of the gait cycle, in terms of *R*^2^, RMSE, 95th percentile, and maximal error (Error_Max_).

**Fig. 3:**
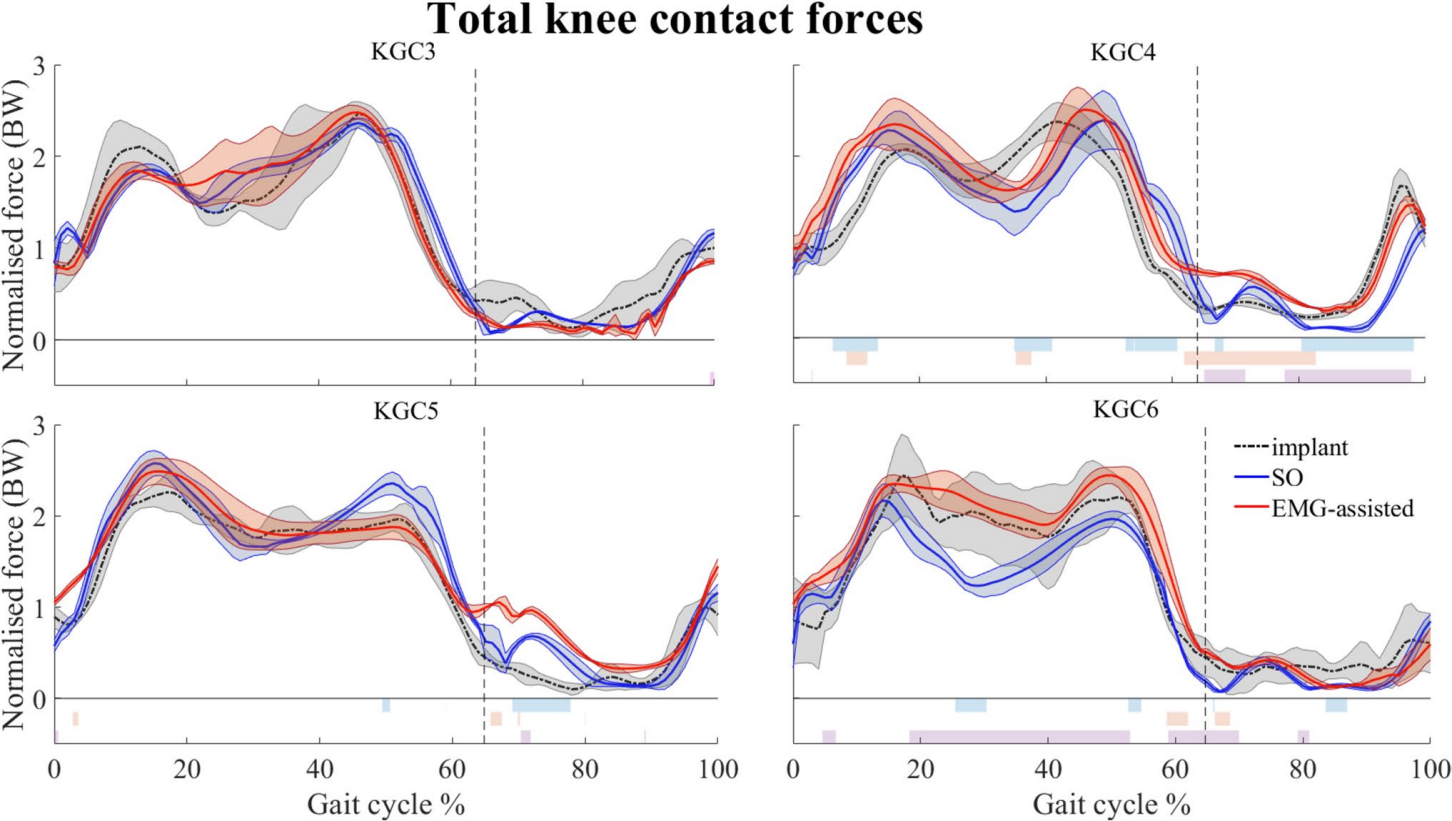
Comparison between predicted and experimental knee joint contact forces. Total knee joint contact forces, expressed in body weight (BW), as recorded by the instrumented implant (*implant*, black dotted), and predicted via the static optimization (blue) and the EMG-assisted approaches (red). Results are reported as mean and standard deviation across the available trials, for each of the KGC competitions. The bars at the bottom identify the parts of the gait cycle where statistical significance was identified by the SPM analysis, while comparing: implant data to static optimization (light blue), implant data to EMG-assisted approach (light red), and static optimization vs EMG-assisted approach (light purple).

For KGC3, the experimental and predicted knee joint contact forces were substantially equivalent. No statistically significant differences were identified by the SPM analysis, in any of the comparisons (i.e., static optimization vs experimental data, EMG-assisted approach vs experimental data. Fig. 3). Contrarily, for the other tested cases, few clusters where the significance threshold was exceeded were observed. In particular, the knee joint contact forces predicted via static optimization differed from the corresponding experimental data in correspondence of (i) the 1st characteristic peak (loading response phase) for KGC4, (ii) the valley (single-leg support phase) for both KGC4 and KGC6, and (iii) the 2nd characteristic peak (pre-swing) for KGC4, KGC5, and KGC6. For the EMG-assisted approach, statistically significant differences were found around the toe-off and, only for KGC4, in correspondence of the 1st peak and valley (Fig. 3).

For KGC6, the SPM analysis further revealed differences in the knee joint contact force profiles predicted by the two approaches during the single-leg support phase (i.e., the static optimization approach underpredicted the values estimated via the EMG-assisted approach, which well approximated the real values) and—in part—during the swing phase of the gait cycle. The latter was observed also for KGC4 and KGC5.

## Discussion

In this work, two common approaches to resolve the muscle redundancy problem, namely the static optimization (hypothesizing optimal muscle control) method and the EMG-assisted approach were employed to perform biomechanical simulations of gait and estimate the knee joint contact forces during an overground walking task, in four elder subjects implanted with an instrumented total knee prosthesis [33]. The main aim was to compare the two approaches against one another and to the experimental recordings, to quantitatively assess their predictive accuracy by means of various metrics (e.g., *R*^2^, RMSE, and SPM analysis).

Overall, the two methods produced similar results, which well approximated the real values, as highlighted by the generally high *R*^2^ values and low RMSEs (*R*^2^ > 0.82, RMSE < 0.56 BW, Tables 2 and 3). This was true for all patients and trials and is in line with previous works [16, 39, 46], thus corroborating the idea that adult individuals free from neuromuscular disorders likely tend to walk adopting a strategy to minimize the metabolic cost. In general, the EMG-assisted method better performed, compared to the classical static optimization approach, both when considering the entire gait cycle (stance and swing phases) and the stance phase only. In the present work, the average RMSE across the stance phase were lower than those reported by Bennett and colleagues [39], for both the SO and EMG-assisted simulations (RMSE_SO_ = 286.1 N vs 422 N, RMSE_EMGa_ = 260 N vs 342 N, considering KGC4-6 for fair comparisons). Of note, a different objective function was implemented in [39], for the EMG-assisted approach in CEINMS.

For two subjects (KGC5 and KGC6) who exhibited atypical knee joint contact force profiles, i.e., characterized by the absence of the second peak (in correspondence of the push off) or a substantial constant loading (barely noticeable valley during the weight acceptance phase), the EMG-assisted method proved able to better approximate the experimental recordings. The static optimization method, on the other hand, did not capture such abnormalities, resulting in *typical* knee joint contact force profiles. This is not surprising, per se. There is evidence in the literature showing that the two approaches may produce different estimates in clinical populations [7]. It is plausible that the two subjects in question adopted a suboptimal muscle control strategy, perhaps due to the fear of falling or feeling of instability and weakness.

This work has some limitations. First, the number of analyzed subjects and trials was limited. This is due to the lack of freely accessible datasets where experimental data from instrumented joint implants are shared alongside medical imaging, surface EMG, and motion capture data, which is essential for such a validation exercise. Moreover, not all trials could be used, as previously highlighted in [39], due to poor quality of the EMG signals. Alternative methods to reconstruct missing EMG data may be employed to compensate for this issue [31], yet the lack of comprehensive datasets remains. Second, all models were generated using a previously validated pipeline [14], starting from the 3D reconstructions of the bones. However, not all datasets did include full lower limb medical images (e.g., of the full pelvis or the feet). Missing components were reconstructed using statistical shape modeling techniques within the MAP Client [35], where mean shape geometries (built off the images of hundreds of adults) were scaled based on the available motion capture markers and morphed—where possible—to accurately represent the available data (e.g., talus) [36]. Nonetheless, any discrepancies between real and reconstructed geometries may have impacted on the definition of the muscle paths and the muscle lever arms. Third, the weights (i.e., α, β, and γ) for the EMG-assisted approach were the same for all participants, potentially limiting the benefits of using the EMG-assisted approach to tune muscle parameters. This choice was motivated by the will to standardize and streamline the procedure. Furthermore, the chosen parameters enabled to well track both the experimental torques and the EMG data for all participants and trials). Last, we assessed the predictive accuracy of the two computational approaches by comparing the resultant contact force at the knee joint estimated by the models and the experimental data recorded by the instrumented implant, but we did not look at the split between medial and lateral sides, which may be important in the study of osteoarthritis. However, a different knee joint mechanism (with two contact points), as in [7, 47], would be needed.

In conclusion, the results of this study support the idea that the static optimization and EMG-assisted approaches can both be used to produce reasonably accurate estimates of knee joint contact forces during walking, in healthy adult participants. However, care should be taken when studying subjects exhibiting atypical walking patterns or patients with neuromuscular disorders, as abnormal features (such as prolonged or anticipated muscle contraction) are likely not captured via static optimization. In such cases, the EMG-assisted approach should be preferred.

## Supplementary Information

Below is the link to the electronic supplementary material.

Supplementary file1 (PDF 490 kb)

## Data Availability

The datasets generated and/or analyzed during the current study are available in the University of Bologna institutional repository under the CC BY 4.0 international license terms at the link https://doi.org/10.6092/unibo/amsacta/7528.

## Abbreviations

CT: Computed tomography
EMG: Electromyography
KGC: Knee grand challenge
MAP: Musculoskeletal atlas project
MIF: Maximal isometric force
MSK: Musculoskeletal
RMSE: Root mean-squared error
SO: Static optimization
SPM: Statistical parametrical mapping
