# Supplementary material for: EMG-Informed Neuromusculoskeletal Simulations Increase the Accuracy of the Estimation of Knee Joint Contact Forces During Sub-optimal Level Walking
Source: Ann Biomed Eng. 2025 Mar 24;53(6):1399–408. doi: 10.1007/s10439-025-03713-2 (PMC12075340; doi:10.1007/s10439-025-03713-2)
Supplement: Supplementary file 1 — Supplementary file1 (PDF 490 kb) [file 10439_2025_3713_MOESM1_ESM.pdf]

SUPPLEMENTARY INFORMATION

This document includes additional data and material produced as part of the work. More specifically, the kinematic errors (resulting from the Inverse Kinematics analysis in OpenSim) and the joint moment tracking errors in CEINMS are reported in Table S1 and Table S2, respectively.

TableS1. Inverse kinematic tracking errors, expressed as mean and standard deviation across the various trials, for all models. The error between corresponding virtual and experimental markers was computed in terms of squared error, RMSE and maximal error ($Error_{Max}$).

	**Squared error (cm^2)**	**RMSE (cm)**	**$Error_{max}$ (cm)**
**KGC3**	0.54 ± 0.10	1.67 ± 0.15	3.07 ± 0.22
**KGC4**	0.52 ± 0.03	1.67 ± 0.05	3.39 ± 0.13
**KGC5**	0.25 ± 0.04	1.15 ± 0.08	2.71 ± 0.17
**KGC6**	0.79 ± 0.16	2.02 ± 0.21	4.07 ± 0.29

TableS2. Joint torques tracking errors, between CEINMS (EMG-assisted approach) and OpenSim (inverse dynamics, considered the method of reference), for all models and degrees-of-freedom. The tracking errors are expressed in terms of RMSE (Nm/kg) and R^2. DOF = degree-of-freedom, PD = plantar-dorsi flexion, FE = flexion-extension, AB = ab-adduction, RT = rotation.

**DOF**	**Metric**	**KGC3**	**KGC4**	**KGC5**	**KGC6**
**Ankle PD**	**R^2**	0.986 ± 0.002	0.997 ± 0.002	0.998 ± 0.001	0.995 ± 0.002
	**RMSE**	0.045 ± 0.003	0.022 ± 0.012	0.022 ± 0.004	0.015 ± 0.007
**Knee FE**	**R^2**	0.998 ± 0.001	0.988 ± 0.005	0.955 ± 0.012	0.999 ± 0.001
	**RMSE**	0.030 ± 0.002	0.123 ± 0.023	0.244 ± 0.041	0.013 ± 0.003
**Hip FE**	**R^2**	0.999 ± 0.001	0.998 ± 0.001	0.999 ± 0.001	0.999 ± 0.001
	**RMSE**	0.008 ± 0.001	0.025 ± 0.004	0.004 ± 0.001	0.013 ± 0.005
**Hip AB**	**R^2**	0.999 ± 0.001	0.994 ± 0.002	0.999 ± 0.001	0.999 ± 0.001
	**RMSE**	0.007 ± 0.002	0.052 ± 0.007	0.003 ± 0.001	0.009 ± 0.002
**Hip RT**	**R^2**	0.983 ± 0.005	0.677 ± 0.056	0.997 ± 0.001	0.979 ± 0.020
	**RMSE**	0.007 ± 0.001	0.073 ± 0.009	0.005 ± 0.001	0.013 ± 0.007
